# Clinical features and treatment outcomes of *Vibrio vulnificus* infection in the coastal city of Ningbo, China

**DOI:** 10.3389/fmicb.2023.1220526

**Published:** 2023-07-04

**Authors:** Jieyi Wang, Xingbei Weng, Yuesong Weng, Quanjun Xu, Yao Lu, Yijun Mo

**Affiliations:** Department of Clinical Laboratory, The First Affiliated Hospital of Ningbo University, Ningbo First Hospital, Ningbo, China

**Keywords:** *Vibrio vulnificus*, sepsis, septic shock, clinical features, laboratory test

## Abstract

**Background:**

*Vibrio vulnificus* is a gram-negative, opportunistic pathogen common to warm waters worldwide. Human *V. vulnificus* infection is rare and typically affects those residing in coastal areas during the summer months, but it causes rapid deterioration and is fatal.

**Methods:**

The medical records of six patients with sepsis caused by *V. vulnificus* infection who were treated at the First Affiliated Hospital of Ningbo University from 2020 to 2022 were retrospectively reviewed. The patient demographics, clinical symptoms, laboratory test results, treatments, and outcomes are summarized.

**Results:**

*Vibrio vulnificus* infection was confirmed by blood or pus culture, 16S ribosomal DNA sequencing, and metagenomic next-generation sequencing. All six patients were male with pre-existing liver diseases and two reported consuming seafood before the onset of symptoms. Of the six patients, four succumbed to the disease, two recovered, and one underwent leg amputation.

**Conclusion:**

*Vibrio vulnificus* infection progresses rapidly and is highly fatal, thus prompt and aggressive treatment is necessary. *Vibrio vulnificus* infection should be considered in older (>40 years) patients with a history of liver disease and recent consumption of seafood or exposure to seawater, especially those residing in coastal areas during the summer months.

## 1. Introduction

*Vibrio vulnificus* is a gram-negative, halophilic, alkaliphilic, and pathogenic marine bacterium commonly found in shellfish and plankton worldwide, particularly in warmer waters (Hollis et al., 1976; Chuang et al., 1989; Baker-Austin and Oliver, 2020). Human infections of *V. vulnificus* mainly occur by consumption of contaminated raw seafood, particularly oysters, or wound exposure to marine environments (Wickboldt and Sanders, 1983; Centers for Disease Control and Prevention, 1993; Bross et al., 2007; Baker-Austin and Oliver, 2020). The incidence of *V. vulnificus* infection is higher in residents of cities by warm coastal areas. *Vibrio vulnificus* is an opportunistic pathogen and infection is highly lethal. The primary characteristics of *V. vulnificus* infection include rapid onset of fever, chills, nausea, abdominal pain, hemorrhagic bullous lesions, cellulitis, and septic shock (Bross et al., 2007; Oliver, 2015; Beatty et al., 2017; Liang et al., 2021). High-risk underlying conditions for *V. vulnificus* infection include liver disease, alcoholism, diabetes, malignancy, immune disorders, and renal disease (Oliver, 2015; Yu et al., 2017; Baker-Austin and Oliver, 2020). Ningbo is an important coastal port city in China and residents consume relatively large amounts of seafood, thereby creating a greater risk for fatal Vibrio infection, especially in the summer months. The aim of this retrospective study is to summarize the demographic characteristics, clinical symptoms, laboratory test results, treatments, and outcomes of patients with *V. vulnificus* infection.

## 2. Methods

### 2.1. Clinical data

The medical records of six patients diagnosed with *V. vulnificus* infection and treated at the First Affiliated Hospital of Ningbo University (Ningbo, China) from 2020 to 2022 were retrospectively reviewed. Patient demographics, clinical symptoms, laboratory test results, antimicrobial therapies, and clinical outcomes were retrieved from electronic medical records.

### 2.2. Laboratory tests

C-reactive protein, procalcitonin, prothrombin time, international normalized ratio, activated partial thromboplastin time, D-dimer, cardiac troponin I, routine blood examinations, and biochemical parameters were assessed with standard equipment and commercial kits.

### 2.3. Identification methods of *Vibrio vulnificus*

*Vibrio vulnificus* isolates were identified in pus or blood samples by pathogen culture, sequencing of 16S ribosomal DNA (rDNA), and/or metagenomic next-generation sequencing (mNGS).

#### 2.3.1. Pathogen culture

Clinical specimens of blood and pus were cultured on Columbia blood agar medium at 35°C and identified by matrix-assisted laser desorption/ionization time-of-flight mass spectrometry (bioMérieux, Marcy-l’Étoile, France).

#### 2.3.2. 16S rDNA sequencing

Sequences of 16S rDNA are highly conserved within the same genus but vary among genera and species. Therefore, 16S rDNA sequencing can be used to accurately identify bacterial isolates and discover novel strains (Woo et al., 2008). In this study, 16S rDNA sequencing of the isolates was conducted by General Biol (Chuzhou, China).

#### 2.3.3. mNGS

mNGS of gene fragments is a highly efficient, rapid, and unbiased high-throughput technology for detection of pathogens in clinical samples (Liu et al., 2022). In this study, mNGS of one blood sample was conducted by Dian Diagnostics Group Co., Ltd (Hangzhou, China).

## 3. Results

### 3.1. Clinical manifestation

Six patients with *V. vulnificus* infections were diagnosed and treated at the First Affiliated Hospital of Ningbo University from 2020 to 2022. All patients were male with a median age of 57 (range, 41–67) years and all had symptoms of sepsis on admission. In the cases of six patients, none of these patients reported contact with seawater. However, it is noteworthy that one patient had a history of exposure to tilapia before admission. One patient presented with fever and reported eating raw mud snails (Bullacta exarate) 5 days before admission and one was admitted after consuming seafood, while the other three reported no knowledge of the mode of *V. vulnificus* infection. All patients resided in Ningbo, a major port city with a local culinary tradition of seafood consumption. Notably, all six patients had pre-existing hepatic disease, including viral hepatitis B cirrhosis and/or alcoholic cirrhosis, and two had diabetes (Table 1).

**Table 1:** Demographic and clinical features of 6 patients with *Vibrio vulnificus* infection.

Characteristic	Patient number					
	1	2	3	4	5	6
Age	51	56	67	41	66	58
Sex	Male	Male	Male	Male	Male	Male
Underlying illness	Alcoholic cirrhosis	Hepatitis B cirrhosis, diabetes	Hepatitis B cirrhosis	Hepatitis B	Alcoholic cirrhosis	Alcoholic cirrhosis, hepatitis B, diabetes
Risk factors	Consumption of raw mud snail	Unclear	Consumption of shrimp	Exposure to tilapia	Unclear	Unclear
Sample	Blood	Blood	Pus	Pus	Blood	Blood
Identification method	pathogen culture + mNGS	pathogen culture +16S rDNA	pathogen culture +16S rDNA	pathogen culture +16S rDNA	pathogen culture +16S rDNA	pathogen culture +16S rDNA
Clinical outcome	Recovered	Death after hospital discharge	Death after hospital discharge	Recovered	Death within 24 h of admission	Death after hospital discharge

As shown in Table 2, common symptoms of *V. vulnificus* infection include fever, local swelling, and erythema. Skin lesions were mainly localized to the distal extremities and most commonly involved the lower limbs. Five patients presented with increased localized skin temperature, four with hemorrhagic bullae and osteofascial compartment syndrome, and one with chest pain and acute myocardial infarction as the primary manifestations. Gastrointestinal symptoms included nausea, vomiting, and diarrhea, and rapidly progressed to sepsis or septic shock.

**Table 2:** Clinical features of *V. vulnificus* infection.

Clinical features	Patient number					
	1	2	3	4	5	6
Most obvious location	Left lower extremity	Left lower extremity	Both lower limbs	Right upper extremity	Both lower limbs	Both lower limbs
Fever	+	+	+	+	+	+
Chill	+	+	−	−	+	+
Nausea and Vomiting	+	−	−	−	+	−
Diarrhea	−	−	−	−	+	−
Local swelling	+	+	+	+	+	+
Erythema	+	+	+	+	+	+
Hemorrhagic bullae	−	+	+	+	−	+
Pain	+	+	+	+	−	+
Localized skin temperature	↑	↑	↑	↑	/	↑
Septic shock	+	+	+	+	+	+
Chest pain	−	−	−	+	−	−
Acute myocardial infarction	−	−	−	+	−	−
Osteofascial compartment syndrome	+	+	+	+	−	−
Consciousness	+	+	+	+	−	+

### 3.2. Laboratory test results

The initial laboratory test results are presented in Table 3. C-reactive protein and procalcitonin levels were significantly elevated in all six patients. Leukocyte counts were increased in two patients and decreased in two others. Hemoglobin levels were < 100 g/L in two patients. Platelet counts were decreased in all patients, with the lowest value of only 11 × 10^9^/L. All six patients had poor liver function with elevated aspartate transferase and decreased albumin levels. Alanine aminotransferase and γ-glutamyltransferase levels were increased in five and four patients, respectively. Additionally, bilirubin, blood urea nitrogen, and creatinine levels were increased in all patients. Cardiac troponin I reached 89 ng/mL in the only patient with acute myocardial infarction. D-dimer levels were significantly increased in five patients.

**Table 3:** Laboratory test results.

Laboratory results	Patient number					
	1	2	3	4	5	6
CRP (mg/L)	164.78	74.04	149.43	168.19	116.46	174.12
PCT (ng/mL)	3.26	97.17	34.96	37.18	>100	33.73
WBC (×10^9^/L)	14.61	13.01	5.18	9.68	1.24	3.12
Neutrophils (%)	12.4	11.9	4.2	8.2	0.7	2.5
Hb (g/L)	86	92	114	142	109	116
PLT (×10^9^/L)	27	41	11	71	106	36
ALT (U/L)	18	76	70	76	111	25
AST (U/L)	64	195	233	384	572	65
GGT (U/L)	135	67	93	14	649	327
LDH (U/L)	206	400	/	682	/	171
CK (U/L)	57	209	/	1,514	/	809
CHE (U/L)	1795	3,853	/	3,842	/	2,321
TBIL (μmol/L)	41	135.9	46.11	24.46	32.08	75.67
TP (g/L)	47.1	50.2	36.9	50.5	36.2	46.7
ALB (g/L)	21	27.4	19.2	30.4	17.9	19.8
BUN (mmol/L)	11.16	12.64	17.1	11	9.6	8.6
Cr (μmol/L)	143	248	148	175	197	164
PT (s)	18.3	23.3	26.5	30.4	17.2	19.2
INR	1.6	2.03	2.31	2.77	1.52	1.71
APTT (s)	33.7	42.7	43.2	95.5	47.8	38.5
DD (ng/mL)	1880	2,834	3,043	324	716	3,055
cTnI (ng/mL)	<0.025	<0.025	3.136	89.653	0.053	0.027

ALB, albumin; ALT, alanine aminotransferase; APTT, activated partial thromboplastin time; AST, aspartate aminotransferase; BUN, blood urea nitrogen; CHE, cholinesterase; CK, creatine kinase; Cr, creatinine; CRP, C-reactive protein; cTnI: cardiac troponin I; DD, D-dimer; GGT, gamma-glutamyl transpeptidase; Hb, hemoglobin; INR, international normalized ratio; LDH, lactate dehydrogenase; PCT, procalcitonin; PLT, platelet count; PT, prothrombin time; TBIL, total bilirubin; TP, total protein; WBC, white blood cell count.

### 3.3. Confirmation of *Vibrio vulnificus*

Standard microbiological methods (pathogen culture, 16S rDNA sequencing, and mNGS) were used to confirm the presence of *V. vulnificus*. Briefly, blood samples were inoculated in standard aerobic/anaerobic bottles containing Columbia blood agar medium and cultured at 35°C, while pus was directly cultured in the medium. *Vibrio vulnificus* isolates were identified using the Vitek® MS microbial identification system (bioMérieux). Antimicrobial susceptibility testing was performed using the microdilution method with a VITEK® 2 Compact system (bioMérieux), and the results are shown in Table 4. *Vibrio vulnificus* was confirmed in the blood or pus specimens of five patients by 16S rDNA sequencing and one blood sample by mNGS.

**Table 4:** Minimum inhibitory concentrations of antimicrobial agents against the 6 *V. vulnificus* isolates.

Drug (μg/mL)	Patient number					
	1	2	3	4	5	6
AAM	/	/	/	<2	<2	<2
AMC	2	2	/	/	/	/
AMK	8	/	4	/	16	8
AMP	<2	<2	<2	<2	<2	<2
CIP	<0.25	<0.25	<0.25	<0.25	1	1
CSL (mm)^*^	/	30	/	/	/	/
CXM	64	/	/	/	/	/
FEP	<1	<1	<1	<1	<1	<1
FOX	/	/	32	/	/	/
GM	4	/	<1	<1	4	<1
IPM	<1	<1	<1	<1	<1	<1
LVX	<0.25	<0.25	<0.25	<0.25	1	1
SXT	<20	<20	<20	<20	>320	<20
TGC	<0.5	<0.5	<0.5	<0.5	<0.5	<0.5
TZP	<4	<4	<4	<4	/	/

AAM, ampicillin-sulbactam; AMC, amoxicillin-clavulanic acid; AMK, amikacin; AMP, ampicillin; CIP, ciprofloxacin; CSL, cefoperazone-sulbactam; CXM, cefuroxime; FEP, cefepime; FOX, cefoxitin; GM, gentamicin; IPM, imipenem; LVX, levofloxacin; SXT, trimethoprim-sulfamethoxazole; TGC, tigecycline; TZP, piperacillin-tazobactam.

*The antibiotic susceptibility testing for cefoperazone-sulbactam in patient 2 was performed using the Kirby-Bauer test.

### 3.4. Treatment and clinical outcome

All patients had an underlying liver disease and, although all received antibiotic therapy on admission, four died of septic shock, as the onset of *V. vulnificus* infection is quite rapid. For instance, a 66-year-old patient with alcoholic cirrhosis was admitted to the intensive care unit (ICU) with a diagnosis of severe sepsis shock, which was treated with cefoperazone-sulbactam and furacilin, but yet died at 24 h after admission.

Of three patients hospitalized with sepsis, one reported seafood ingestion 3 days prior to admission. The antimicrobial agents received by all patients are shown in Table 5. One patient underwent amputation and two were treated by incision and decompression. However, the prognoses of these three patients were poor and the families eventually chose to voluntarily withdraw treatment. Two patients survived *V. vulnificus* infection, including one with alcoholic cirrhosis who consumed raw mud snails 5 days before admission. *Vibrio vulnificus* infection was highly suspected based on clinical presentation before confirmation by pathogen culture. The second surviving patient, aged 41 years, with hepatitis B developed acute myocardial infarction while hospitalized elsewhere. The patient had a history of exposure to tilapia before admission. He was transferred to our hospital and underwent right upper limb amputation for *V. vulnificus* infection (Table 5). Both patients received aggressive treatment and were discharged in good condition.

**Table 5:** Treatments of 6 patients with *V. vulnificus* infection.

Treatments	Patient number					
	1	2	3	4	5	6
CRRT	+	+	+	−	+	−
Hormone therapy	−	−	+	+	−	+
Transfusion	+	+	+	+	+	+
Tracheal cannula	+	+	+	+	+	−
Incision and decompression	+	+	+	+	−	−
Amputation	−	+	−	+	−	−
Antibiotic therapy	Cefoperazone-sulbactam + levofloxacin	Piperacillin-tazobactam + moxifloxacin	Cefoperazone-sulbactam + levofloxacin	Meropenem + levofloxacin	Cefoperazone-sulbactam + furacilin	Cefoperazone-sulbactam + moxifloxacin

CRRT, continuous renal replacement therapy.

## 4. Discussion and conclusion

*Vibrio vulnificus* is a virulent bacterium that inhabits warm, brackish seawater, and human infection is quite rare. However, recent studies have suggested that increased surface water temperatures due to global warming have enhanced the distribution of *V. vulnificus* (Lin et al., 2021). The epidemiology of *V. vulnificus* infection is challenging to determine accurately, as surveillance and reporting systems vary among countries and regions. *Vibrio vulnificus* is prevalent in coastal regions of China, and some studies have reported that the detection rate of *V. vulnificus* in retail seafood in the southeastern coastal region of China is around 20% (Chen et al., 2009). A local study conducted in Beilun, a district of Ningbo, detected *V. vulnificus* mainly in shellfish sold in July and August (Wo et al., 2022).

*Vibrio vulnificus* causes fatal infections in humans through two main routes: primary sepsis resulting from the ingestion of raw or undercooked seafood and necrotizing wound infections due to exposure to warm seawater with high concentrations of *V. vulnificus*. The susceptibility of the host to *V. vulnificus* infection is critical to disease progression. This infection typically occurs in patients with chronic liver disease, especially alcoholic cirrhosis and chronic hepatitis B or C infection. Additionally, patients with chronic conditions, such as alcoholism, diabetes, malignancy, immune disorders, end-stage renal disease, rheumatoid arthritis, and hemochromatosis, are at higher risks for *V. vulnificus* infection (Johnston et al., 1985; Barton and Acton, 2009; Oliver, 2015; Yu et al., 2017; Baker-Austin and Oliver, 2020). Patients with *V. vulnificus* infections mostly present clinically with acute fever, chills, shock, extremity skin bullae, and skin/muscle necrosis. The disease develops extremely rapidly, with an average incubation period of only 16 h for wound infections and 26 h for sepsis. The mortality rate of wound infections is approximately 15% and over 50% for primary sepsis (Bross et al., 2007; Baker-Austin and Oliver, 2020). Interestingly, males and elderly patients appear to have a much greater risk for severe infection than women and younger patients. Notably, some reports suggest that men are six times more likely to be infected with *V. vulnificus* than women, possibly because of the higher prevalence of cirrhosis in men and older age groups (>40 years) (Lee et al., 2013; Baker-Austin and Oliver, 2018, 2020). These findings are also consistent with the characteristics of the cases described in this study.

*Vibrio vulnificus* infections are characterized by rapid onset of symptoms that can develop into fatal systemic infections within days. The devastating nature of these infections suggests that the pathogenicity is a multifactorial and complex phenomenon involving many virulence factors, such as capsular polysaccharides, lipopolysaccharide (LPS), multifunctional auto-processing repeats-in-toxin, phospholipase A2, hemolysin/cytolysin, and iron acquisition systems (Jones and Oliver, 2009; Choi and Choi, 2022). Studies as early as the 1980s have reported a correlation between the virulence and capsular polysaccharide of *V. vulnificus*. For example, Yoshida et al. (1985) found that the capsular materials of *V. vulnificus* conveyed resistance to serum bactericidal and antiphagocytic activities and were highly lethal in mice and strongly invasive in the subcutaneous tissues of guinea pigs. Another study reported that loss of the capsule was accompanied by decreased virulence, hydrophilicity, and serum resistance (Wright et al., 1990). Shock is a common feature of *V. vulnificus* infection likely due to the presence of LPS, which is a known pyrogen that is reported to elicit a small cytokine response in mice and the subsequent release of tumor necrosis factor α (Powell et al., 1997). These toxins are critical for *V. vulnificus* to avoid phagocytosis, promote host tissue damage, disseminate into the bloodstream and other organs, and cause septic shock (Choi and Choi, 2022).

Generally, *V. vulnificus* strains are susceptible to most antibiotics used in veterinary and clinical medicine. The antibiotics currently recommended for treatment of *V. vulnificus* infection are doxycycline, cephalosporin (e.g., ceftazidime), fluoroquinolone (e.g., levofloxacin, ciprofloxacin, or gatifloxacin), and trimethoprim-sulfamethoxazole plus aminoglycosides (Elmahdi et al., 2016). However, due to the excessive use of antibiotics in agriculture, aquaculture, and clinical settings, antibiotic resistance has become concerning for various strains of bacteria, including *V. vulnificus*. Resistance of *V. vulnificus* to common antibiotics has reached alarming levels in many countries (Cabello, 2006; Heng et al., 2017). Since the fatality rate of *V. vulnificus* infection increases with the delay of disease onset and timing of antibiotic administration, it is crucial to administer appropriate antibiotic therapy immediately when *V. vulnificus* infection is suspected (Bross et al., 2007; Daniels, 2011). A study by Klontz et al. (1988), of 62 patients found that the mortality rate of *V. vulnificus* infection increased with the time to antibiotic administration from 33% at 24 h after admission to 63 and 100% at 48–72 and > 72 h, respectively.

Many patients require aggressive supportive care in the ICU in addition to antibiotics. *Vibrio vulnificus* infection complicated with hemorrhagic bullous skin lesions/necrotizing fasciitis, skin/soft tissue infections involving two or more limbs, and primary septicemia has been linked to an increased risk of death in the ICU. Patients treated with surgery plus antibiotics, especially those who receive a timely surgical evaluation within 24 h of admission, may have a better prognosis (Chen et al., 2010; Kuo Chou et al., 2010). Hence, proactive and timely wound care is critical. Meanwhile, surgical debridement, incision, and drainage of abscesses, and even amputation have been shown to reduce mortality (Bross et al., 2007). In addition to the above, hyperbaric oxygen therapy has been reported to be helpful in the treatment of *V. vulnificus* infection. Tamura et al. found that hyperbaric oxygen had a remarkable therapeutic effect on *V. vulnificus* infection provoked by its injection into mouse footpads (Tamura et al., 2012). And Kot et al. reported that after using hyperbaric oxygen therapy as an adjunctive treatment for patients with *V. vulnificus* infection, the general and local condition improved, septic shock resolved, and the inflammatory parameters decreased (Kot and Lenkiewicz, 2022). Unfortunately, our hospital does not have a hyperbaric chamber and cannot give hyperbaric oxygen therapy to patients with *V. vulnificus* infection.

In conclusion, *V. vulnificus* infections are easily confused with skin and subcutaneous tissue infections and sepsis caused by other pathogens, but require different targeted antibiotic therapies and treatment methods. *Vibrio vulnificus* infection should be considered in older (>40 years) patients with a history of liver disease and recent consumption of seafood or exposure to seawater, especially those residing in coastal areas during the summer months. Prompt and aggressive antibiotic therapy, intensive treatment of shock and symptoms of multi-organ dysfunction, surgical debridement, incision, and drainage of abscesses, and even amputation may be necessary.

## Data availability statement

The datasets presented in this study can be found in online repositories. The names of the repository/repositories and accession number(s) can be found below: NCBI – OQ975991-OQ975995.

## Ethics statement

The studies involving human participants were reviewed and approved by The Ethics Committee of Ningbo First Hospital. Written informed consent for participation was not required for this study in accordance with the national legislation and the institutional requirements.

## Author contributions

JW, XW, YW, QX, YL, and YM contributed to the study conception and design. Material preparation, data collection, and analysis were performed by JW and YM. The first draft of the manuscript was written by JW and all authors commented on previous versions. All authors contributed to the article and approved the submitted version.

## Funding

This work was financially supported by a grant from the Zhejiang Province Basic Public Welfare Research Program Project (grant no. LGC21H200002).

## Conflict of interest

The authors declare that the research was conducted in the absence of any commercial or financial relationships that could be construed as a potential conflict of interest.

## Publisher’s note

All claims expressed in this article are solely those of the authors and do not necessarily represent those of their affiliated organizations, or those of the publisher, the editors and the reviewers. Any product that may be evaluated in this article, or claim that may be made by its manufacturer, is not guaranteed or endorsed by the publisher.
